# Impact of acute kidney injury and renal recovery status in deceased donor to kidney transplant outcome: results from the Thai national transplant registry

**DOI:** 10.1038/s41598-023-47928-6

**Published:** 2023-11-22

**Authors:** Nuttasith Larpparisuth, Supanit Nivatvongs, Kajohnsak Noppakun, Adisorn Lumpaopong, Cholatip Pongskul, Peenida Skulratanasak

**Affiliations:** 1 Division of Nephrology, Department of Medicine, Faculty of Medicine Siriraj Hospital, Mahidol University, 2 Prannok Road, Bangkok Noi, Bangkok, 10700 Thailand; 2 Organ Donation Center, Thai Red Cross Society, Bangkok, Thailand; 3 Department of Surgery, Faculty of Medicine, Chulalongkorn University and King Chulalongkorn Memorial Hospital, Bangkok, Thailand; 4 Division of Nephrology, Department of Internal Medicine, Chiang Mai University, Chiang Mai, Thailand; 5 Division of Pediatric Nephrology, Department of Pediatrics, Phramongkutklao Hospital and College of Medicine, Bangkok, Thailand; 6 Division of Nephrology, Department of Internal Medicine, Faculty of Medicine, Khon Kaen University, Khon Kaen, Thailand

**Keywords:** Kidney, Renal replacement therapy

## Abstract

The influence of acute kidney injury (AKI) and renal recovery in deceased donor (DD) on long-term kidney transplant (KT) outcome has not previously been elucidated in large registry study. Our retrospective cohort study included all DDKT performed in Thailand between 2001 and 2018. Donor data was reviewed case by case. AKI was diagnosed according to the KDIGO criteria. Renal recovery was defined if DD had an improvement in AKI to the normal or lower stage. All outcomes were determined until the end of 2020. This study enrolled 4234 KT recipients from 2198 DD. The KDIGO staging of AKI was as follows: stage 1 for 710 donors (32.3%), stage 2 for 490 donors (22.3%) and stage 3 for 342 donors (15.6%). AKI was partial and complete recovery in 265 (17.2%) and 287 (18.6%) before procurement, respectively. Persistent AKI was revealed in 1906 KT of 990 (45%) DD. The ongoing AKI in DD significantly increases the risk of DGF development in the adjusted model (HR 1.69; 95% CI 1.44–1.99; *p* < 0.001). KT from DD with AKI and partial/complete recovery was associated with a lower risk of transplant loss (log-rank *P* = 0.04) and recipient mortality (log-rank *P* = 0.042) than ongoing AKI. KT from a donor with ongoing stage 3 AKI was associated with a higher risk of all-cause graft loss (HR 1.8; 95% CI 1.12–2.88; *p* = 0.02) and mortality (HR 2.19; 95% CI 1.09–4.41; *p* = 0.03) than stage 3 AKI with renal recovery. Persistent AKI, but not recovered AKI, significantly increases the risk of DGF. Utilizing kidneys from donors with improving AKI is generally safe. KT from donors with persistent AKI stage 3 results in a higher risk of transplant failure and recipient mortality. Therefore, meticulous pretransplant evaluation of such kidneys and intensive surveillance after KT is recommended.

## Introduction

Acute kidney injury (AKI) is the common complication in deceased donors (DD) who suffered hemodynamic instability and hormonal change after brain death^1,2^. In the general population, AKI is a well-known risk factor for the further development of cardiovascular problems and chronic kidney disease (CKD), known as the AKI-CKD continuum^3,4^. The increase in donor terminal serum creatinine (SCr) is also integrated into the US kidney donor profile index (KDPI) as a harmful factor for transplant survival^5^. Several recent studies revealed an association between DD AKI status and the development of delayed graft function (DGF)^6–9^, but there was no definitive impact of donor AKI on long-term graft survival^8–10^. However, some studies still showed conflicting results^6,7,11^. Most of these large studies were conducted in developed western countries that had several differences in context with developing countries^6,9–11^.

The shortage of DD numbers is a major barrier to KT in Thailand, thus the utilization of kidneys from DD with AKI can effectively represent the donor pool^12^. Data from the Thai Transplant Registry during 2016–2020 found that 30–40% of DD had terminal SCr > 1.5 mg/dL^13^, which is higher than western countries^14^. DDs in our country are younger, have a lower prevalence of diabetes mellitus (DM), hypertension (HT), and cerebrovascular accident (CVA) than in developed countries, but we have a higher prevalence of donor hypotension, the need for cardiopulmonary resuscitation (CPR), and relatively longer cold ischemic time (CIT)^13,15,16^. The authentic incidence of donor AKI in Thailand or in the developing country model had not been previously assessed. Several DD with AKI in Thailand received an effective resuscitation and subsequent recovery before procurement. The outcome of improving AKI in DD is not elucidated in the large registry data. The present study reviewed our local transplant data to determine the impact of AKI and recovery status on transplant and recipient outcome in Thailand.

## Methods

We conducted a retrospective cohort study, which enrolled all deceased donor kidney-only transplants (DDKTs) in Thailand between 1 January 2001 and 31 December 2018. The national Thai data were recovered from the database of the Thai Red Cross Society for donor data and the Thai Transplant Registry for recipient characteristics and KT outcomes. This study was approved by the Ethics Committees of the Thai Transplant Society and the Siriraj Hospital Faculty of Medicine, Mahidol University, Bangkok, Thailand (COA No. Si 394/2020), with a waiver of informed consent. Study data were collected from electronic database without violation of patients’ identities. The study was performed in accordance with international guidelines for human research protection.

DD data was reviewed case by case for demographic data, hemodynamic profiles, need for vasopressors and CPR, change in kidney function, and urine volume throughout the pre-donation period. Donor hypotension was defined as a systolic blood pressure lower than 90 mm Hg for a consecutive 2-h period. The estimated glomerular filtration rate (eGFR) of the best SCr was calculated with the Chronic Kidney Disease Epidemiology Collaboration equation (CKD-EPI) for adult donors^17^ and the revised Schwartz formula for pediatric donors^18^. The kidney donor profile index was also calculated based on the Thai Eq.^16^. The diagnosis and staging of AKI were defined by the KDIGO guideline; both SCr and urine output criteria^19^. There was no DD that required dialysis in our registry because the kidney was generally discarded.

Renal recovery from AKI in DD was also reviewed in all cases and defined based on the change in the KDIGO SCr level criteria. 'Partial recovery' was defined if serum creatinine decreased to the lower stage of AKI but not to the baseline level. 'Complete recovery' was defined in the case of reduction of SCr to the baseline value. Demographic data, comorbidities, duration of dialysis of KT recipients (KTRs) were gathered. The outcome of interest for the analysis included all-cause transplant loss, death-censored graft loss, and death by the end of 2020.

### Statistical methods

The donor, recipient and transplant factors are shown as percentage (%) number and percentage (%), or mean ± standard deviation (SD) for the normally distributed data and as median and interquartile range for nonnormally distributed data. Comparison of categorical variables was analyzed using Chi-square tests, while continuous variables were compared using Student's t test or the Mann–Whitney *U* test, as appropriate. A two-tailed *p*-value < 0.05 was statistically significant.

Kaplan–Meier analysis was used to compare survival between groups and was reported as logarithmic rank *p* value. Cox proportional-hazard regression analysis—adjusted for significant donor factors (age, sex, diabetes, hypertension, death from CVA, weight and height), recipient factors (age, sex, diabetes, duration of dialysis, panel reactive antibody) and transplant factors (CIT, HLA mismatches)—was used to assess the associations between each variable and transplant or recipient survival. All analyzes were performed using PASW Statistics for Windows (version 18.0; SPSS Inc., Chicago, Ill., USA).

### Ethics declarations

This study was approved by the Ethics Committees of the Thai Transplant Society and the Siriraj Hospital Faculty of Medicine, Mahidol University, Bangkok, Thailand (COA No. Si 394/2020), with a waiver of informed consent. Study data were collected from electronic database without violation of patients’ identities. The study was performed in accordance with international guidelines for human research protection.

## Results

After applying the exclusion criteria, 4234 DDKTs of 2198 donors were enrolled (Supplement Fig. 1). The baseline characteristics of all DDKTs and kidney donors stratified by donor AKI status are shown in Tables 1 and 2, respectively. The median follow-up duration for the KT recipients was 74.9 ± 53.6 months. The mean age of the DD and KT recipients was 37 ± 14.2 and 43.7 ± 13.5 years, respectively. Male is the preferred sex for both DD (80.2%) and recipients (61%). The prevalences of donor HT, donor DM and donor deaths from CVA were 11.9%, 2.5% and 25.4%, respectively. Recipients of kidney DD with AKI had a significantly higher height (161.2 ± 14.8 vs. 160.4 ± 16.1; *p* = 0.04) but other comparable profiles. CIT was significantly higher in KT from the AKI donor (19.4 ± 5.4 vs. 18.8 ± 5.4; *p* = 0.001).

**Table 1:** Baseline characteristics KT recipients and transplant profiles in the cohort stratified by presence or absence of AKI in DD.

Parameter	All DDKTs (n = 4234)	DDKTs from donor with AKI (n = 2969)	DDKTs from donor without AKI (n = 1265)	*P* value
Recipient age (years)	43.7 ± 13.5	44 ± 13.6	43.2 ± 13.3	0.10
Recipient male sex (%)	61	61.4	60	0.39
Recipient height (cm)	161.2 ± 14.8	161.6 ± 14.2	160.4 ± 16.1	0.04
Recipient weight (kg)	58.6 ± 16.3	58.8 ± 17	58.1 ± 14.4	0.21
Recipient dialysis vintage (years)	4.7 ± 3.4	4.7 ± 3.3	4.7 ± 3.5	0.81
Recipient follow up duration (years)	6.2 ± 4.5	6.16 ± 4.4	6.4 ± 4.6	0.53
Recipient DM (%)	11.8	12.1	11.8	0.48
HLA mismatch (number)	2 (1–3)	2 (1–3)	2 (1–3)	0.92
PRA (%)	0 (0)	0 (0)	0 (0)	0.74
Cold ischemic time (hours)	19.2 ± 5.4	19.4 ± 5.4	18.8 ± 5.4	0.001
Induction therapy				
No antibody induction (%)	31.6	30.4	34.4	0.228
IL-2 receptor antagonist (%)	53.9	56.8	54	
Antithymocyte globulin (%)	10.1	10.6	9	
Initial calcineurin inhibitors				
Tacrolimus (%)	87.2	87.5	86.6	0.498
Cyclosporin (%)	12.8	12.5	13.4	

*DDKT* deceased donor kidney transplantation, *AKI* acute kidney injury, *DM* diabetes mellitus, *HLA* human leukocyte antigen, *PRA* panel reactive antibody, *IL-2* interleukin-2.

**Table 2:** Baseline characteristics of kidney donors in the cohort stratified by presence or absence of AKI in deceased donor.

Parameter	All donors (n = 2198)	DD with AKI (n = 1542)	DD without AKI (n = 656)	*P* value
Donor age (years)	37 ± 14.2	37.1 ± 13.8	36.7 ± 15.2	0.52
Donor male sex (%)	80.1	83.1	72.9	**< 0.001**
Donor height (cm)	165.6 ± 9.2	166.3 ± 8.6	164.1 ± 10.3	**< 0.001**
Donor weight (kg)	64 ± 11.8	64.8 ± 11.5	62 ± 12.1	**< 0.001**
Donor with DM (%)	2.5	2.7	2.1	0.471
Donor with HT (%)	11.9	12.8	9.8	**0.041**
Death from CVA (%)	25.4	26.9	22	**0.015**
Donor best SCr (mg/dL)	0.93 ± 0.4	0.99 ± 0.45	0.79 ± 0.22	**< 0.001**
Donor eGFR calculated from best SCr (ml/min/1.73 m^2^)	99.3 ± 25.8	96.1 ± 26.3	106.8 ± 22.7	**< 0.001**
Donor maximum SCr (mg/dL)	1.79 ± 1.15	2.15 ± 1.19	0.93 ± 1.19	**< 0.001**
Donor last SCr (mg/dL)	1.57 ± 1.12	1.87 ± 1.12	0.85 ± 0.24	**< 0.001**
Donor hypotension (%)	76.5	79.8	68.8	**< 0.001**
Donor received CPR (%)	14.9	16.1	12.2	**0.019**
Donor received dopamine infusion (%)	76.8	76.7	76.8	0.955
Donor received noradrenaline infusion (%)	46.9	49.6	40.4	** =< 0.001**
Donor received adrenaline infusion (%)	22	25.5	13.7	** =< 0.001**
Donor Thai KDPI > 80% (%)	21.6	22.8	18.8	**0.034**

*DD* deceased donor, *AKI* acute kidney injury, *DM* diabetes mellitus, *HT* hypertension, *SCr* serum creatinine, *CPR* cardiopulmonary resuscitation, *KDPI* kidney donor profile index.

Significant values are in [bold].

**Figure 1:**
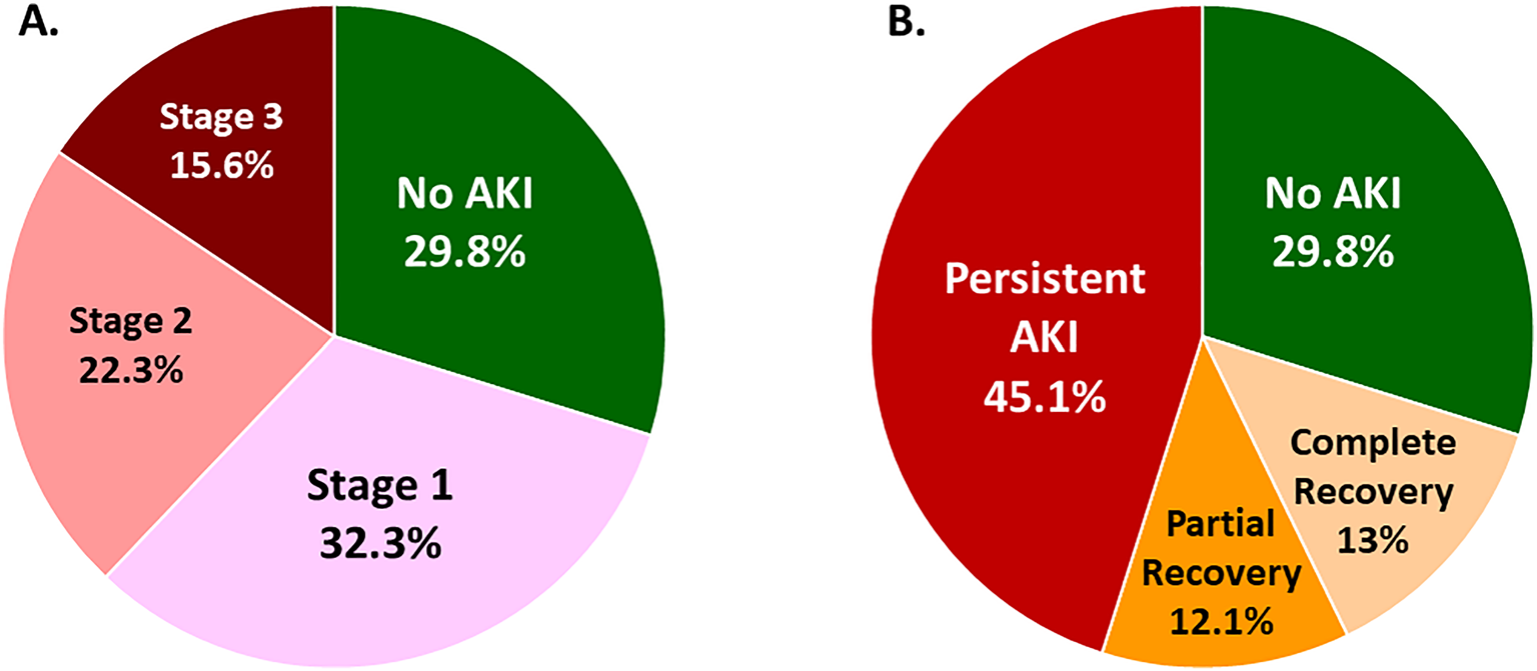
Staging of AKI and recovery status in the entire deceased donor. (**A**) Prevalence of AKI and its staging, (**B**) recovery status of AKI.

The prevalence of AKI in our DD was 70.2% (1542 donors). The majority (70.1%; 2969 recipients) of DDKT also received kidneys from DD with AKI. Most DD had normal baseline kidney function (mean eGFR from best serum creatinine, 99.3 ± 25.8 ml/min/1.73 m^2^). According to the KDIGO guideline, the AKI staging was determined by serum creatinine criteria, urine output criteria and both criteria in 1420 (64.6%), 15 (0.7%) and 107 (4.9%) DD, respectively. For AKI staging in DD, 710 (32.3%) DD had AKI stage 1490 (22.3%) developed AKI stage 2, and 342 (15.6%) suffered from AKI stage 3 (Fig. 1A). The maximum serum creatinine ≥ 3 mg/dL and ≥ 4 mg/dL was revealed in 257 (11.7%) and 113 (5.1%) DD, respectively.

DD with AKI had higher weight (64.8 ± 11.5 vs. 62 ± 12.1; *p* < 0.001), height (166.3 ± 8.6 vs. 164.1 ± 10.3; *p* < 0.001), prevalence of HT (12.8% vs. 9.8%; *p* = 0.041), CVA (26.9% vs. 22%; *p* = 0.015) and Thai KDPI > 80% than those without AKI (22.8% vs. 18.8%; *p* = 0.034). DD who had AKI also had higher best serum creatinine (SCr) (0.99 ± 0.45 vs. 0.79 ± 0.22; *p* < 0.001), which resulted in lower best estimated glomerular filtration rate (eGFR) (96.1 ± 26.3 vs. 106.8 ± 22.7; *p* < 0.001). Noradrenaline and adrenaline were prescribed more for DD with AKI (Table 2). The recovery status of DD AKI was determined in all cases before procurement. AKI without renal recovery (Persistent AKI) was revealed in 1906 KT of 990 DD, while 265 (17.2%) and 287 (18.6%) had partial and complete recovery of kidney function before procurement, respectively (Fig. 1B). DDs with persistent AKI were older and received more vasopressors and CPR compared to AKI with complete recovery. However, we found no significant differences in baseline characteristics between AKI with partial recovery and persistent AKI, except higher prevalence of adrenaline infusion in persistent AKI. (Supplement Table 1).

### Effect of donor AKI on delayed graft function (DGF) and early loss of graft

**Figure 2:**
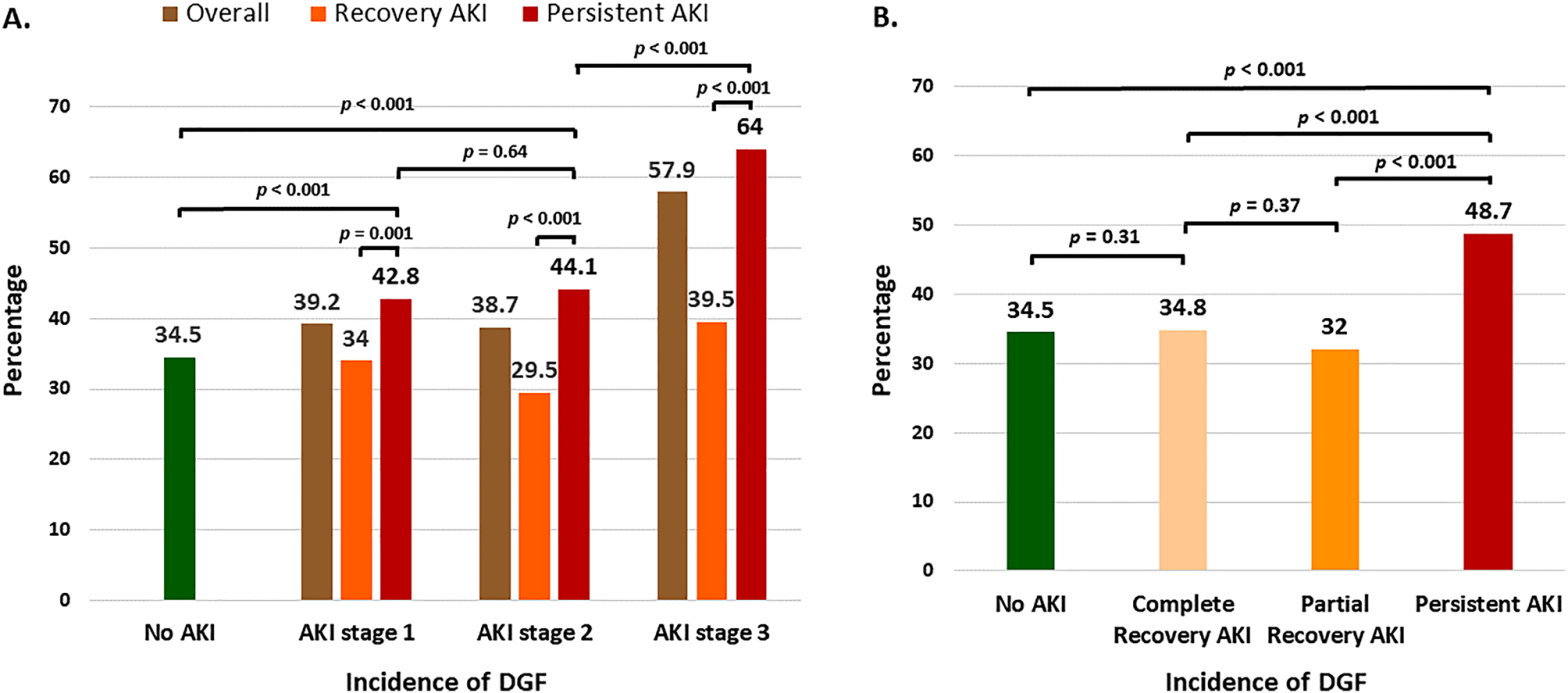
Incidence of delayed graft function. (**A**) Comparison between no AKI and each AKI staging stratified by recovery status, (**B**) comparison between AKI with complete recovery, partial recovery, and persistent AKI.

The incidence of DGF in the overall cohort was 40.6%. KT from DD with AKI was associated with a higher incidence of DGF compared to DD without AKI (43.2% vs. 34.5%; *p* < 0.001). The incidence of DGF is significantly higher in AKI stage 3 than in no AKI, stage 1 and 2 AKI, respectively (57.9% vs. 34.5% vs. 39.2% vs. 38.7%; *p* < 0.001) (Fig. 2A). When we took into account renal recovery from AKI in DD, KT from a donor with persistent AKI had significantly higher incidence of DGF compared to AKI with partial or complete recovery, respectively (48.7% vs. 32% vs. 34.8%; *p* < 0.001) (Fig. 2B).

The development of DGF was relatively comparable between KT from DD without AKI and partial (34.5% vs. 32%; *p* = 0.31) or complete recovery (34.5% vs. 34.8%; *p* = 0.97). There was no difference in the incidence of DGF among each staging of DD AKI with partial or complete recovery and those without AKI. Persistent AKI stage 3 in DD resulted in a significantly higher incidence of DGF than AKI stage 1 or 2 (64% vs. 44.1% vs. 42.8%; *p* < 0.001). Stage 1 and 2 of ongoing donor AKI also had a higher incidence of DGF than receiving kidney from donor without AKI (44.1% vs. 42.8% vs. 34.5%; *p* < 0.001). From multivariate analysis, the risk of DGF occurrence was significantly increased when KT from donor with stage 3 AKI (HR 2.41; 95% CI 1.95–2.98; *p* < 0.001) or persistent AKI (HR 1.69; 95% CI 1.44–1.99; *p* < 0.001) (Supplement Table 2).

The early loss of allograft within the first 3 months was revealed in 121 (2.9%) KT. There was no difference in the incidence of early graft loss by donor AKI status (3.1% vs. 2.4%; *p* = 0.22) and by renal recovery status (2.4% without AKI, 2.4% in partial recovery, 3.7% in complete recovery, and 3.2% in persistent AKI; *p* = 0.37).

### Impact of donor AKI on all-cause graft failure

**Figure 3:**
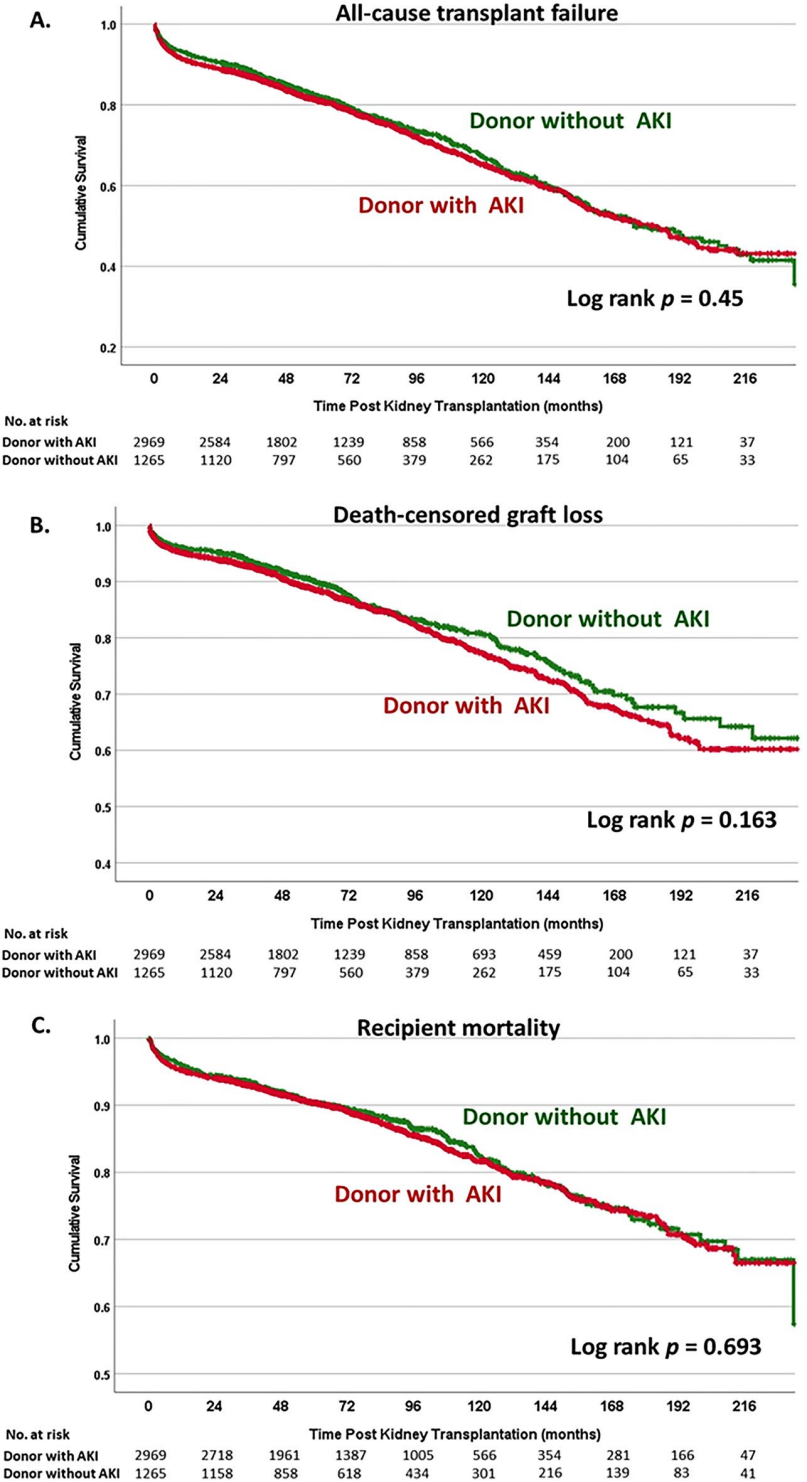
Kaplan–Meier analysis compared between KT from donor with AKI and without AKI. (**A**) All-cause transplant failure, (**B**) death-censored graft loss, and (**C**) mortality.

During the study period with a median follow-up time of 62.1 (IQR 36.3–104.6) months, there were 1164 (27.5%) transplant losses; 673 KT (15.9%) with graft loss and 491 (11.6%) death with functioning graft. Kaplan–Meier analysis showed comparable overall transplant (log-rank *p* = 0.45) and death-censored graft survival (log-rank *p* = 0.16) between KT from DD with and without AKI (Fig. 3A,B). The Cox regression hazard model adjusted for crucial factors revealed no differences in all-cause transplant loss (HR 0.98; 95% CI 0.85–1.14; *p* = 0.81) and death-censored graft failure (HR 1.12; 95% CI 0.92–1.37; *p* = 0.26) by DD AKI status. (Supplement Table 3 and 4).

When focused on AKI staging, Kaplan–Meier analysis did not show substantial differences in all-cause transplant failure (log-rank *P* = 0.39) and death-censored graft loss (log-rank *P* = 0.17) among each staging of AKI staging (Supplement Fig. 2). Multivariate analysis did not demonstrate a significant risk of transplant loss in KTRs who received DD with stage 1 of AKI (HR 0.97; 95% CI 0.81–1.16; *p* = 0.72), 2 (HR 1.02; 95% CI 0.93–1.13; *p* = 0.66), and 3 (HR 0.95; 95% CI 0.88–1.02; *p* = 0.17) compared to KT from donor without AKI. There was also a comparable risk of death-censored graft failure to KT from DD without AKI, with of 1.14 (95% CI 0.9–1.45), 1.24 (95% CI 0.96–1.61), 0.92 (95% CI 0.67–1.26) for stage 1, 2 and 3, respectively (Supplement Tables 3 and 4).

**Figure 4:**
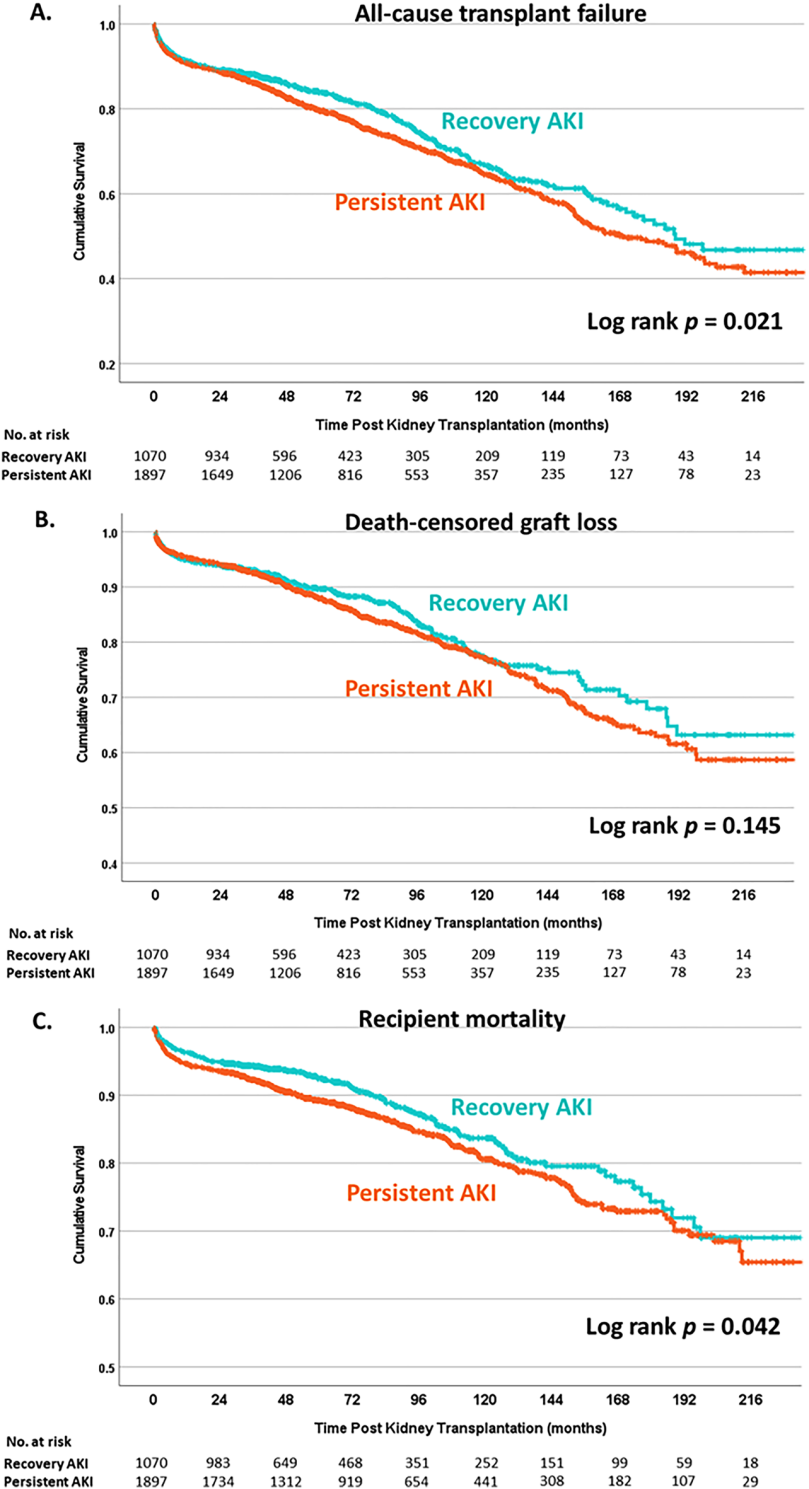
Kaplan–Meier analysis compared between KT from donor with persistent AKI and AKI with renal recovery. (**A**) All-cause transplant failure, (**B**) death-censored graft loss, and (**C**) mortality.

Furthermore, we analyzed renal recovery status before DD with AKI. KT from DD with AKI and partial/complete recovery was associated with a lower risk of transplant loss (log-rank *P* = 0.04) than ongoing AKI, however, the difference in death-censored graft survival was not evidenced (log-rank *P* = 0.14) (Fig. 4A,B). We also cooperated with AKI staging to determine renal recovery status to determine transplant outcome. For stage 1 and stage 2, the ongoing AKI did not increase the risk of transplant failure with HR of 0.94 (95% CI 0.75–1.19) and 1.12 (95% CI 0.86–1.46), respectively. However, there was a trend towards inferior transplant survival in KT of the donor with persistent AKI stage 3, compared to stage 3 AKI with partial or complete recovery (log-rank *P* = 0.098) (Supplement Fig. 3A). The Cox regression hazard model revealed a higher risk of all-cause transplant loss (HR 1.8; 95% CI 1.12–2.88; p = 0.02) in persistent stage 3 AKI compared to the renal recovery group (Table 3).

**Table 3:** Factors associated with all-cause transplant failure in KT from deceased donor with AKI stage 3.

Variables	Univariate analysis			Multivariate analysis		
	HR	95% CI	*p-*value	HR	95% CI	*p-*value
Donor age	0.992	0.969–1.015	0.489			
Donor sex (female)	1.027	0.591–1.784	0.924			
Donor height	1.012	0.98–1.046	0.462			
Donor weight	0.991	0.975–1.008	0.292			
Donor diabetes	**4.745**	**2.196–10.253**	** < 0.001**	**4.058**	**1.972–8.351**	** < 0.001**
Donor hypertension	**1.666**	**0.911–3.048**	**0.098**	**1.761**	**1.136–2.728**	**0.011**
Donor CVA	1.157	0.599–2.237	0.664			
Donor best SCr	0.931	0.587–1.476	0.761			
Donor eGFR from best SCr	0.997	0.985–1.009	0.625			
Donor CPR	0.769	0.47–1.259	0.296			
Donor Thai KDPI	1.002	0.983–1.021	0.864			
Recovery status of donor AKI						
AKI with recovery	Ref			Ref		
Persistent AKI	**1.652**	**1.013–2.696**	**0.044**	**1.797**	**1.123–2.875**	**0.015**
Recipient age	0.998	0.984–1.013	0.819			
Recipient gender (female)	0.777	0.534–1.131	0.188			
Recipient diabetes	1.361	0.823–2.253	0.23			
HLA mismatch	0.99	0.988–1.015	0.815			
Transplant cold ischemic time	1.028	0.994–1.063	0.106			

*HR* Hazard ratio, *CVA* cerebrovascular accident, *SCr* serum creatinine, *CPR* cardiopulmonary resuscitation, *AKI* acute kidney injury, *KDPI* kidney donor profile index.

Significant values are in [bold].

### Impact of donor AKI on recipient survival

During the study period, there were 611 deaths (14.4%); 491 (11.6%) died with a functioning graft and 120 (2.8%) passed away after graft loss. The primary known cause of mortality in our population was infection (52.7%), followed by cardiovascular disease (22.1%) and cancer (7.7%). These rates were comparable between KT from DD with AKI and those without AKI (*p* = 0.853). Kaplan–Meier analysis showed comparable survival of KTR between KT of DD with and without AKI (log-rank *p* = 0.69) (Fig. 3C). KT from DD with AKI and partial/complete recovery was associated with a lower risk of recipient mortality (log-rank *P* = 0.042) than ongoing AKI (Fig. 4C). The Cox regression hazard model revealed no differences in recipient survival (HR 0.92; 95% CI 0.76–1.11; *p* = 0.39) according to the status of DD AKI. There was no significant difference in mortality risk between each stage of AKI, with HR of 0.93 (95% CI 0.75–1.15), 1.01 (95% CI 0.80–1.28), 0.79 (95% CI 0.60–1.05) for stage 1, 2 and 3, respectively (Supplement Table 5). Kaplan–Meier analysis revealed that KTRs who received DD with persistent AKI tended to have lower survival than DD who recovered from AKI (log-rank *P* = 0.066) (Supplement Fig. 3B). KT from a donor with ongoing stage 3 AKI was associated with a higher risk of mortality than stage 3 AKI with renal recovery (HR 2.19; 95% CI 1.09–4.41; *p* = 0.03) from multivariate analysis (Table 4).

**Table 4:** Factors associated with mortality in patients who received kidney from deceased donor with AKI stage 3.

Variables	Univariate analysis			Multivariate analysis		
	HR	95% CI	*p-*value	HR	95% CI	*p-*value
Donor age	0.976	0.945–1.007	0.132			
Donor sex (female)	1.298	0.612–2.752	0.496			
Donor height	1.066	1.019–1.115	0.005			
Donor weight	0.969	0.943–0.995	0.02	0.986	0.968–1.003	0.103
**Donor diabetes**	**4.082**	**1.325–12.576**	**0.014**	**4.003**	**1.604–9.99**	**0.03**
Donor hypertension	0.764	0.32–1.824	0.544			
Donor CVA	0.437	0.187–1.022	0.056			
Donor best SCr	1.143	0.618–2.112	0.67			
Donor eGFR from best SCr	1.013	0.995–1.031	0.151			
Donor CPR	0.679	0.347–1.33	0.259			
Donor Thai KDPI	1.030	1.003–1.057	0.028	1.003	0.993–1.013	0.533
**Recovery status of donor AKI**						
AKI with recovery	Ref			Ref		
**Persistent AKI**	**2.055**	**0.993–4.252**	**0.052**	**2.187**	**1.085–4.405**	**0.029**
**Recipient age**	**0.998**	**0.984–1.013**	**0.819**	**1.028**	**1.009–1.047**	**0.004**
Recipient gender (female)	0.725	0.431–1.219	0.225			
Recipient diabetes	1.468	0.791–2.724	0.224			
HLA mismatch	0.992	0.986–1.014	0.804			
Transplant cold ischemic time	1.023	0.997–1.071	0.332			

*HR* Hazard ratio, *CVA* cerebrovascular accident, *SCr* serum creatinine, *CPR* cardiopulmonary resuscitation, *AKI* acute kidney injury, *KDPI* kidney donor profile index.

Significant values are in [bold].

## Discussion

To increase the number of organs of DD, the use of kidneys from donor with AKI is a noteworthy alternative option. Nevertheless, the effectiveness and safety of such a strategy are still questionable. In this study, we revealed a higher incidence of DGF when KT from a donor with persistent AKI, not for AKI with recovery. We found no association between donor AKI and worse transplant outcome. The sub-analysis revealed no definite impact of donor stage 1 and 2 of AKI, however, KT from DD with persistent AKI stage 3 had significantly higher all-cause graft failure and mortality than AKI with recovery.

The prevalence of donor AKI in our country of 70.2% is much higher than the previous reports from western countries, which are approximately 10–38%^6,9–11^. Despite the healthier donor without the use of the donor after circulatory death, there was a higher incidence of donor hypotension, the need for cardiopulmonary resuscitation and vasopressors, including adrenaline, and a relatively longer cold ischemic time in Thailand^13,15,16,20^. The explanations were lack of designated donor care team, insufficient medical personnel and resources, delay in detection and donation process for potential donor.

The diagnosis of donor AKI in our study was primarily based on SCr criteria, only 0.7% reached only urine output criteria. Since diabetes insipidus is the inevitable consequence after brain death^1^, a reduction in urine volume does not appear in most cases. The results of almost previous studies that diagnosed AKI by individual SCr level are generally reliable^10,11^. We found that AKI is more common in DD who had underlying HT, brain death from CVA, and higher KDPI. This result is not different from the general setting in which patients with comorbid diseases tended to develop AKI during hemodynamic instability^19,21^. Donors with higher BMI, which require a higher volume of fluid resuscitation, were also at risk of the occurrence of AKI^22^. Thus, marginal and large donor needs must be managed with careful attention.

Donor AKI was a significant risk factor for DGF, particularly in the case of stage 3 AKI, as supported by prior studies and a recent systematic review^6,11,23^. Our multivariate analysis confirmed a significant association between donor AKI and the occurrence of DGF. However, the prevalence of DGF in our country is relatively high but comparable to several developing countries^24–26^. The high rate of DGF in Thailand can be attributed to several factors, including a high prevalence of donor AKI, prolonged cold ischemic time, and a shortage of dedicated surgeons and nephrologists for KT care. We observed no significant impact of overall AKI and the staging of AKI on both all-cause transplant loss and death-censored graft loss. These findings are consistent with several previous studies that employed adjusted models and a recent systematic review^8–10,23^. Kidney transplantation from elderly DD with AKI resulted in worse transplant survival. However, our study lacked the statistical power to definitively address this issue, as only 4% of our DD were over the age of 60^27^. Given that the DD in our registry were relatively healthier than the reports in high-income countries, the reversibility of AKI is more probable, resulting in equivalent long-term survival.

One of the most important objectives of our study focused on recovery status of donor AKI. The delay in donor detection and suboptimal initial donor resuscitation in primary care hospitals are the leading cause of DD AKI in our country. However, after proper resuscitation, AKI began to improve in several donors. The outcome of donor AKI with partial recovery was not well elucidated from the large registry data. Previous studies from Korea showed that improving donor AKI had superior graft survival than ongoing, and even without AKI^28^. This might be explained by ischemic preconditioning. On the other hand, a recent study from the French Registry revealed that donor AKI, including both ongoing or recovery groups, had higher risk of graft failure than without AKI^11^. However, our studies showed a favorable outcome for donor AKI with partial or complete recovery, beginning with a comparable incidence of DGF and long-term graft and recipient survival. The inconsistent findings in the French study could be attributed to the younger age of the donors with less comorbidities in the Thai Registry. Generally, almost all recovery AKI is likely a pre-renal cause which had no structural damage to the renal parenchyma. Therefore, from our finding, kidney utilization of kidneys from a donor with improving AKI is generally safe in both short- and long-term outcomes.

In contrast, persistent AKI was associated with inferior transplant survival from Kaplan–Meier analysis; however, this turned out to be non-significance after adjusting with other factors. We also found that ongoing AKI stage 3 had worse transplant and recipient survival than AKI with recovery. With a significantly higher incidence of DGF in persistent stage 3 of AKI, this finding may not be surprising and was consistent with a previous study from the UK^6^. Our earlier studies from the Thai Transplant Registry demonstrated inferior graft and patient survival in the DGF group. KT recipients who experienced DGF, with challenges in adjusting immunosuppression, faced a higher risk of infection, which emerged as the leading cause of death in our recipients^29,30^. We did not support the discard of organs from DD with AKI; however, intensive surveillance of post-transplant infection and complications should be performed.

Our study has several strengths. First, the diagnosis of AKI was established by both SCr level and urine output criteria. Second, all donor data was thoroughly reviewed case by case. Third, we enrolled all DDKTs in a national database to reduce bias. Our data had a relatively high incidence of donor AKI and a low rate of loss to follow-up. Finally, we have nearly complete data of donor, recipient, and transplant parameters and put them all in multivariate analysis to figure out the impact of AKI. However, our study has some limitations. First, we did not have complete data on organ waste, so we did not analyze this issue. Since we have no available pretransplant biopsy, kidneys from a high-KDPI donor with AKI were sometimes discarded or used in a dual KT manner. Second, even though we enrolled all KTs in our country over an 18-year period, the study drew on a relatively smaller sample compared to western countries. However, our data might be more suitable for application in developing countries that have donor and recipient profiles comparable to the Thai population.

## Conclusions

Persistent donor AKI, but not AKI with recovery, significantly increased the risk of DGF. Utilization of kidneys from donors with recovered AKI is generally considered safe. KT from the donor with AKI had an equivalent outcome to those of the donor without AKI. However, KT from donors with persistent AKI stage 3 had a higher risk of graft loss and mortality compared to those with improving AKI. Therefore, we recommend careful evaluation of such kidneys before transplantation and intensive surveillance for infection after KT.

### Supplementary Information

Supplementary Information.

## Data Availability

The datasets analyzed during the current study are not publicly available due to restrictions from the Thai Transplantation Society. The data that support the findings of this study are available from the corresponding author on reasonable request and with permission of the Thai Transplantation Society.
