# Supplementary material for: Impact of acute kidney injury and renal recovery status in deceased donor to kidney transplant outcome: results from the Thai national transplant registry
Source: Sci Rep. 2023 Nov 22;13:20492. doi: 10.1038/s41598-023-47928-6 (PMC10665315; doi:10.1038/s41598-023-47928-6)
Supplement: Supplementary file 1 — Supplementary Information. [file 41598_2023_47928_MOESM1_ESM.pdf]

Title: Impact of Acute Kidney Injury and Renal Recovery Status in Deceased Donor to Kidney Transplant Outcome: Results from the Thai National Transplant Registry.

Authors and Affiliations: Nuttasith Larpparisuth, MD[1]; Supanit Nivatvongs, MD[2,3]; Kajohnsak Noppakun, MD[4]; Adisorn Lumpaopong, MD[5]; Cholatip Pongskul, MD[6]; Peenida Skulratanasak, MD[1]

[1] *Division of Nephrology, Department of Medicine, Faculty of Medicine Siriraj Hospital, Mahidol University, Bangkok, Thailand*

[2] *Organ Donation Center, Thai Red Cross Society*

[3] *Department of Surgery, Faculty of Medicine, Chulalongkorn University and King Chulalongkorn Memorial Hospital, Bangkok, Thailand.*

[4] *Division of Nephrology, Department of Internal Medicine, Chiang Mai University, Chiang Mai, Thailand*

[5] *Division of Pediatric Nephrology, Department of Pediatrics, Phramongkutklao Hospital and College of Medicine, Bangkok, Thailand*

[6] *Division of Nephrology, Department of Internal Medicine, Faculty of Medicine, Khon Kaen University, Khon Kaen, Thailand*

Supplement Table 1: Baseline characteristics of kidney donors who had AKI in the cohort stratified by recovery status

**Parameter**	**AKI with complete recovery** (n = 287)	**AKI with partial recovery** (n = 265)	***P* value** (Complete vs Partial)	**Persistent AKI** (n = 990)	***P* value** (Complete vs Persistent)	***P* value** (Partial vs Persistent)
Donor age (years)	35.2 ± 14.6	37.6 ± 13.2	**0.046**	37.5 ± 13.7	**0.017**	0.89
Donor male sex (%)	78.4	80.8	0.493	85.2	**0.006**	0.081
Donor height (cm)	165.9 ± 8.5	166 ± 7.9	0.932	166.4 ± 8.8	0.394	0.459
Donor weight (kg)	63.4 ± 10.4	64.8 ± 11.7	0.146	65.2 ± 11.7	**0.025**	0.658
Donor with DM (%)	2.1	2.6	0.67	2.8	0.494	0.87
Donor with HT (%)	11.1	14.7	0.211	12.8	0.448	0.42
Death from CVA (%)	23.7	25.7	0.592	28.2	0.132	0.415
Donor best SCr (mg/dL)	0.84 ± 0.19	0.97 ± 0.36	**< 0.001**	1.04 ± 0.5	**< 0.001**	**0.02**
Donor eGFR calculated from best SCr (ml/min/1.73 m^2)	105.4 ± 20.8	96.7 ± 25.6	**< 0.001**	93.3 ± 27.3	**< 0.001**	0.071
Donor maximum SCr (mg/dL)	1.44 ± 0.42	2.23 ± 0.83	**< 0.001**	2.33 ± 1.34	**< 0.001**	0.247
Donor last SCr (mg/dL)	0.91 ± 0.23	1.55 ± 0.56	**< 0.001**	2.25 ± 1.3	**< 0.001**	**< 0.001**
Donor hypotension (%)	74.6	83	**0.015**	80.4	**0.032**	0.336
Donor received CPR (%)	12.9	11.7	0.67	18.2	0.036	0.012
Donor received dopamine infusion (%)	72.8	75.8	0.416	78.1	0.063	0.439
Donor received noradrenaline infusion (%)	43.6	60	< 0.001	48.6	0.133	**< 0.001**
Donor received adrenaline infusion (%)	16.7	18.5	0.586	29.9	**< 0.001**	**< 0.001**
Donor Thai KDPI > 80% (%)	19.2	24.9	0.103	23.3	0.136	0.593

Abbreviations: DD, deceased donor; AKI, acute kidney injury; DM, diabetes mellitus; HT, hypertension; SCr, serum creatinine; CPR, cardiopulmonary resuscitation; KDPI, kidney donor profile index

Supplement Table 2: Factors associated with the development of delayed graft function from univariate and multivariate analysis

Variables	Univariate analysis			Multivariate analysis		
	**HR**	**95% CI**	***p*-value**	**HR**	**95% CI**	***p*-value**
Donor age	1.014	1.005-1.023	0.003	1.012	1.005-1.019	0.001
Donor sex (female)	1.197	0.975-1.469	0.086			
Donor height	1.002	0.989-1.016	0.76			
Donor weight	1.007	0.999-1.014	0.088	1.009	1.002-1.015	0.007
Donor diabetes	1.001	0.636-1.575	0.997			
Donor hypertension	1.118	0.868-1.44	0.39			
Donor CVA	1.011	0.785-1.301	0.934			
Donor best SCr	1.083	0.77-1.524	0.646			
Donor eGFR from best SCr	0.998	0.993-1.004	0.57			
Donor CPR	1.183	0.974-1.438	0.09			
Donor Thai KDPI	1.003	0.995-1.010	0.503	1.004	1.001-1.008	0.013
Donor AKI	1.281	1.096-1.496	0.002	1.311	1.125-1.527	< 0.001
Donor AKI staging						
No AKI	Ref.			Ref.		
Stage 1 AKI	1.199	0.997-1.442	0.054			
Stage 2 AKI	1.057	0.862-1.297	0.593			
Stage 3 AKI	2.329	1.857-2.921	< 0.001	2.408	1.947-2.977	< 0.001
Recovery status of donor AKI						
No AKI	Ref.			Ref.		
AKI with recovery	0.894	0.737-1.085	0.256			
Persistent AKI	1.634	1.378-1.938	< 0.001	1.692	1.438-1.991	< 0.001
Recipient age	1.008	1.003-1.014	0.002	1.009	1.003-1.014	0.002
Recipient gender (female)	0.832	0.723-0.957	0.01	0.833	0.724-0.958	0.01
Recipient diabetes	1.272	1.031-1.571	0.025	1.264	1.024-1.56	0.029
HLA mismatch	1.01	0.987-1.016	0.783			
Transplant cold ischemic time	1.081	1.066-1.095	< 0.001	1.081	1.067-1.095	< 0.001

Abbreviations: HR, Hazard ratio; CVA, cerebrovascular accident; SCr, serum creatinine; CPR, cardiopulmonary resuscitation; AKI, acute kidney injury; KDPI, kidney donor profile index

Supplement Table 3: Factors associated with all-cause transplant failure from univariate and multivariate analysis

Variables	Univariate analysis			Multivariate analysis		
	**HR**	**95% CI**	***p*-value**	**HR**	**95% CI**	***p*-value**
Donor age	1.015	1.007-1.023	< 0.001	1.010	1.005-1.016	< 0.001
Donor sex (female)	1.092	0.905-1.318	0.357			
Donor height	0.99	0.978-1.002	0.119	0.993	0.986-1.000	0.043
Donor weight	0.996	0.989-1.003	0.284			
Donor diabetes	1.993	1.401-2.835	< 0.001	1.944	1.411-2.679	< 0.001
Donor hypertension	1.277	1.020-1.599	0.033			
Donor CVA	1.615	1.285-2.031	< 0.001	1.487	1.273-1.738	< 0.001
Donor best SCr	0.93	0.698-1.238	0.618			
Donor eGFR from best SCr	0.993	0.988-0.998	0.007	0.996	0.993-0.998	< 0.001
Donor CPR	0.968	0.802-1.17	0.739			
Donor Thai KDPI	0.995	0.988-1.002	0.16			
Donor AKI	0.982	0.846-1.140	0.811			
Donor AKI staging						
No AKI	Ref.			Ref.		
Stage 1 AKI	0.968	0.81-1.157	0.723			
Stage 2 AKI	1.022	0.928-1.127	0.656			
Stage 3 AKI	0.947	0.876-1.023	0.167			
Recovery status of donor AKI						
No AKI	Ref.			Ref.		
AKI with recovery	0.912	0.754-1.103	0.343			
Persistent AKI	1.012	0.86-1.192	0.882			
Recipient age	0.996	0.991-1.001	0.104			
Recipient gender (female)	0.966	0.845-1.105	0.615			
Recipient diabetes	1.271	1.031-1.571	0.025	1.214	0.993-1.484	0.059
HLA mismatch	1.010	0.995-1.020	0.21			
Transplant cold ischemic time	1.027	1.015-1.039	< 0.001	1.026	1.014-1.038	< 0.001

Abbreviations: HR, Hazard ratio; CVA, cerebrovascular accident; SCr, serum creatinine; CPR, cardiopulmonary resuscitation; AKI, acute kidney injury; KDPI, kidney donor profile index

Supplement Table 4: Factors associated with death-censored graft failure from univariate and multivariate analysis

Variables	Univariate analysis			Multivariate analysis		
	HR	95% CI	*p*-value	HR	95% CI	*p*-value
Donor age	1.027	1.017-1.038	< 0.001	1.026	1.016-1.037	< 0.001
Donor sex (female)	1.173	0.916-1.502	0.205			
Donor height	0.982	0.967-0.998	0.023	0.98	0.968-0.992	0.001
Donor weight	0.994	0.985-1.004	0.253			
Donor diabetes	2.317	1.496-3.587	< 0.001	2.24	1.455-3.448	< 0.001
Donor hypertension	1.478	1.112-1.964	0.007	1.453	1.099-1.921	0.009
Donor CVA	1.957	1.464-2.616	< 0.001	1.920	1.439-2.562	< 0.001
Donor best SCr	0.743	0.485-1.136	0.17			
Donor eGFR from best SCr	0.987	0.981-0.994	< 0.001	0.991	0.987-0.995	< 0.001
Donor CPR	1.066	0.836-1.358	0.606			
Donor Thai KDPI	0.99	0.982-0.999	0.24	0.991	0.982-0.999	0.033
Donor AKI	1.124	0.919-1.374	0.255			
Donor AKI staging						
No AKI	Ref.			Ref.		
Stage 1 AKI	1.142	0.901-1.449	0.272			
Stage 2 AKI	1.242	0.961-1.606	0.098			
Stage 3 AKI	0.922	0.672-1.263	0.611			
Recovery status of donor AKI						
No AKI	Ref.			Ref.		
AKI with recovery	1.053	0.816-1.359	0.69			
Persistent AKI	1.173	0.942-1.46	0.153			
Recipient age	0.976	0.97-0.982	0.104	0.977	0.97-0.983	< 0.001
Recipient gender (female)	0.946	0.794-1.127	0.533			
Recipient diabetes	1.271	1.031-1.571	0.025	1.214	0.993-1.484	0.059
HLA mismatch	1.011	0.994-1.022	0.25			
Transplant cold ischemic time	1.03	1.014-1.045	< 0.001	1.03	1.015-1.046	< 0.001

Abbreviations: HR, Hazard ratio; CVA, cerebrovascular accident; SCr, serum creatinine; CPR, cardiopulmonary resuscitation; AKI, acute kidney injury; KDPI, kidney donor profile index

Supplement Table 5: Factors associated with post-transplant mortality from univariate and multivariate analysis

Variables	Univariate analysis			Multivariate analysis		
	**HR**	**95% CI**	***p*-value**	**HR**	**95% CI**	***p*-value**
Donor age	1.001	0.989-1.013	0.873			
Donor sex (female)	1.156	0.886-1.509	0.285			
Donor height	1.005	0.987-1.022	0.61			
Donor weight	0.992	0.981-1.003	0.139			
Donor diabetes	1.675	0.997-2.812	0.051			
Donor hypertension	0.967	0.695-1.345	0.84			
Donor CVA	1.157	0.832-1.61	0.385			
Donor best SCr	1.082	0.755-1.551	0.668			
Donor eGFR from best SCr	1.000	0.994-1.007	0.916			
Donor CPR	0.894	0.684-1.167	0.408			
Donor Thai KDPI	1.004	0.994-1.014	0.453	1.005	1.001-1.008	0.007
**Donor AKI**	0.897	0.73-1.103	0.302			
**Donor AKI staging**						
No AKI	Ref.			Ref.		
Stage 1 AKI	0.833	0.649-1.069	0.151			
Stage 2 AKI	0.943	0.718-1.237	0.671			
Stage 3 AKI	0.843	0.614-1.157	0.29			
**Recovery status of donor AKI**						
No AKI	Ref.			Ref.		
AKI with recovery	0.803	0.615-1.049	0.108	0.792	0.607-1.032	0.084
Persistent AKI	0.938	0.748-1.177	0.581			
Recipient age	1.026	1.018-1.034	< 0.001	1.027	1.019-1.035	< 0.001
Recipient gender (female)	0.990	0.819-1.196	0.915			
Recipient diabetes	1.359	1.052-1.755	0.02	1.353	1.05-1.745	0.02
HLA mismatch	1.010	0.993-1.023	0.28			
Transplant cold ischemic time	1.028	1.012-1.045	< 0.001	1.028	1.012-1.045	< 0.001

Abbreviations: HR, Hazard ratio; CVA, cerebrovascular accident; SCr, serum creatinine; CPR, cardiopulmonary resuscitation; AKI, acute kidney injury; KDPI, kidney donor profile index

Supplement Figure 1: Consort flow diagram of the study

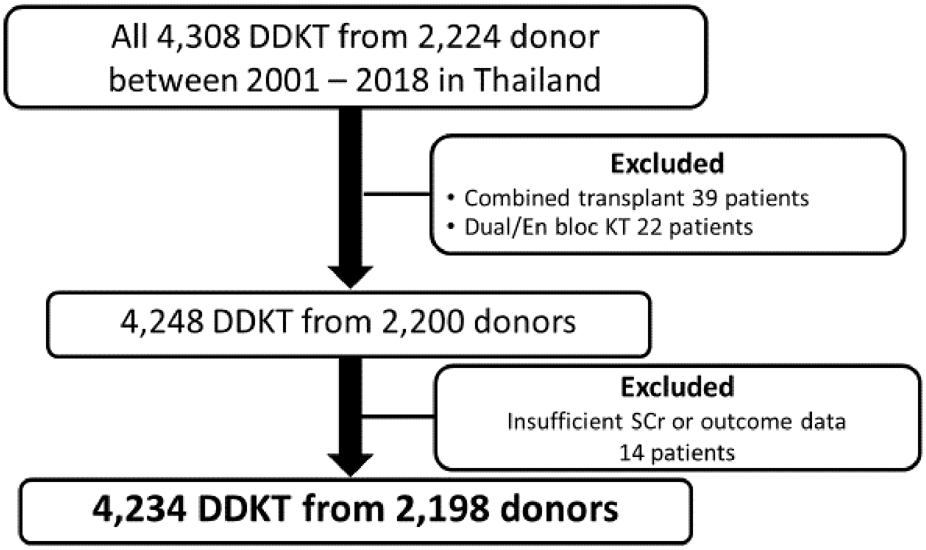

Supplement Figure 2: The Kaplan-Meier analysis compared between KT from deceased donor without AKI and with each stage of AKI. A) All-cause transplant failure and B) death-censored graft loss

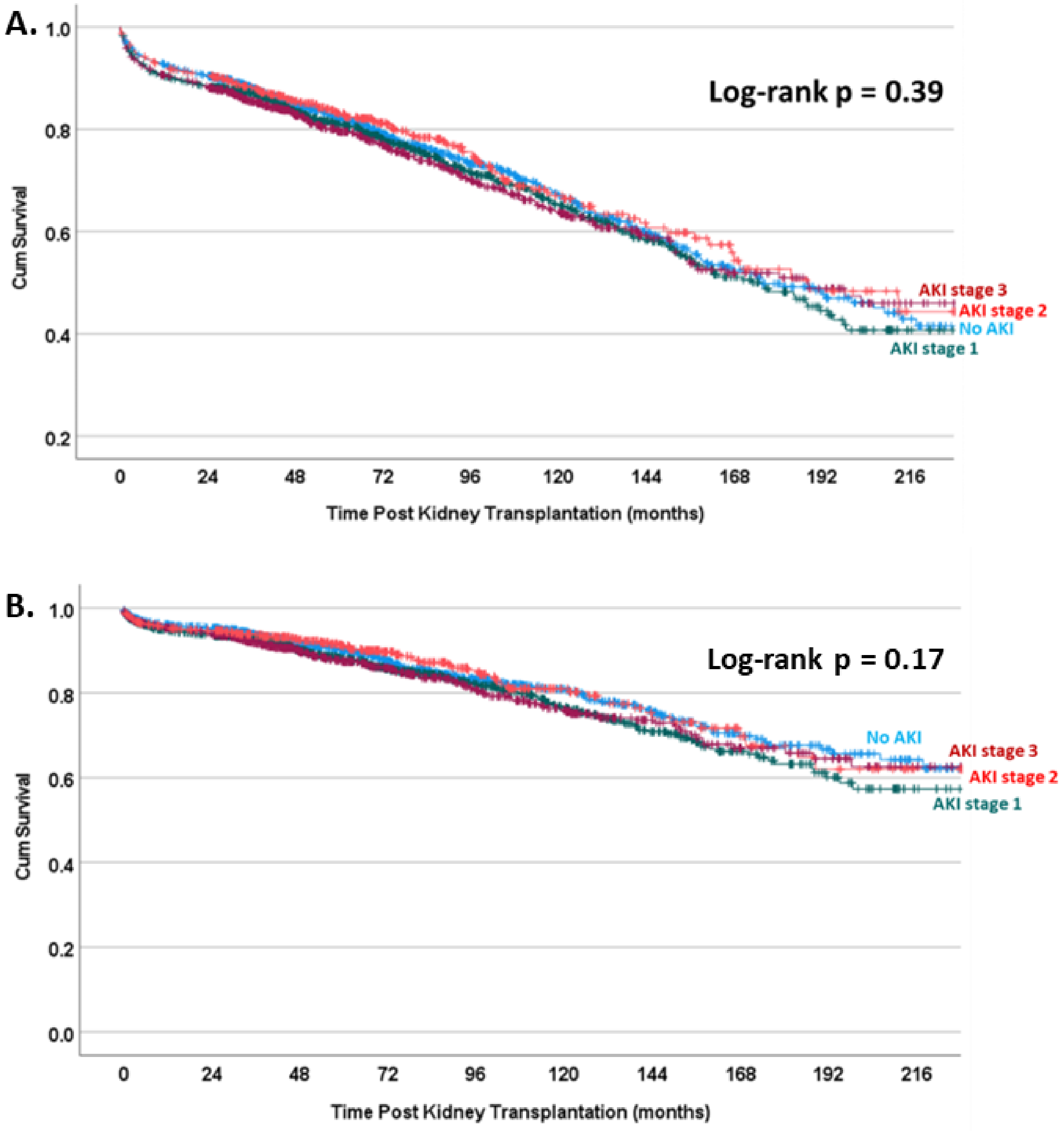

Supplement Figure 3: The Kaplan-Meier analysis compared renal recovery status in KT from donor who had AKI stage 3. A) All-cause transplant failure and B) mortality

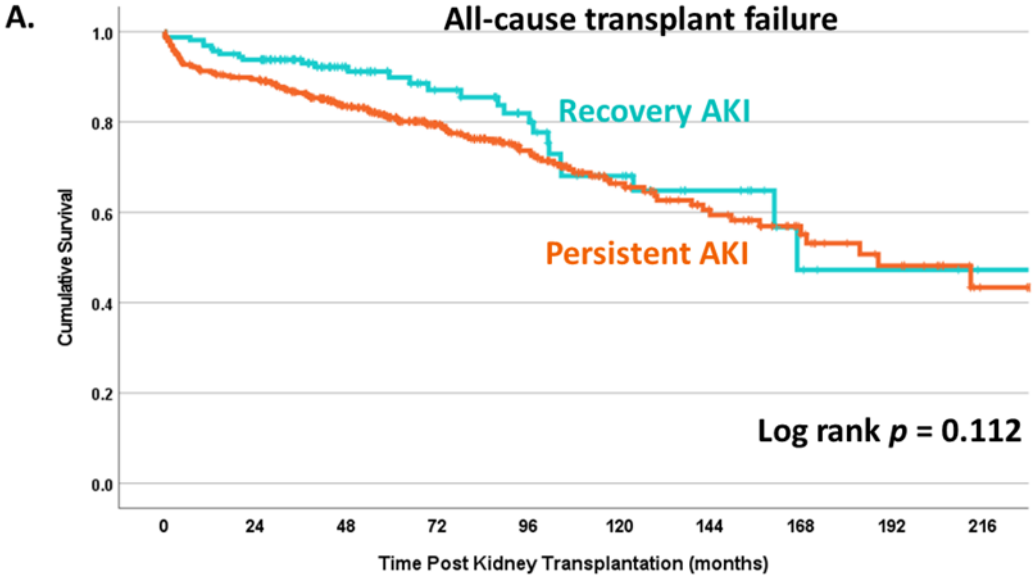

No. at risk

Recovery AKI	163	151	90	58	42	27	15	6	4	1
Persistent AKI	493	432	333	212	131	77	51	28	18	6

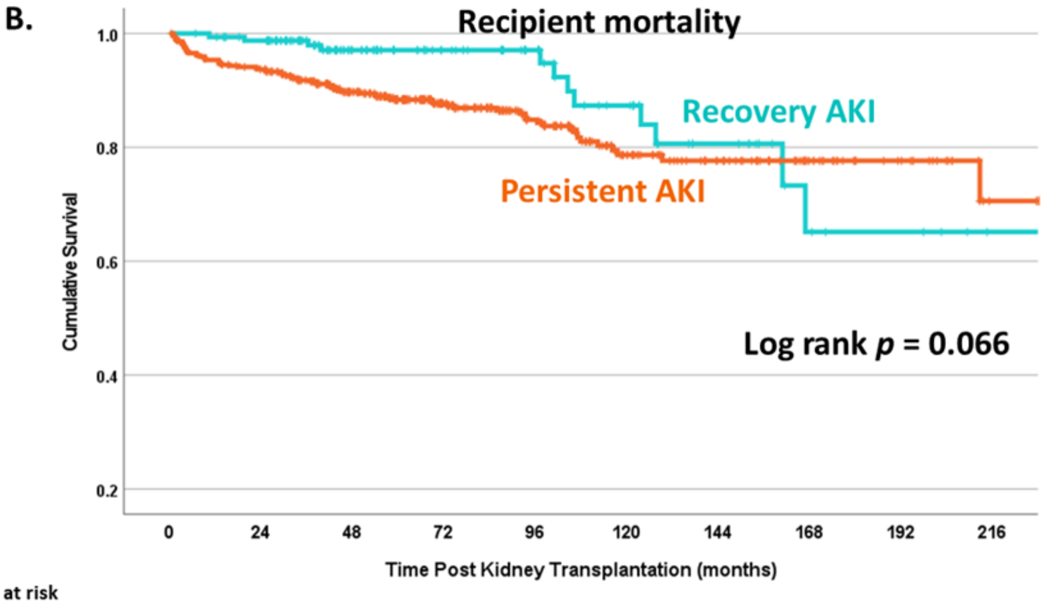

No. at risk

Recovery AKI	163	156	92	63	47	33	19	9	7	2
Persistent AKI	493	453	358	231	153	91	64	37	22	7
